# Predicting disease progression in progressive cerebellar ataxias: integrating clinical, imaging, fluid, genetic, and digital biomarkers

**DOI:** 10.3389/fneur.2026.1921210

**Published:** 2026-08-17

**Authors:** Yingxu Hao, Xianjun Hao

**Affiliations:** 1 The First Affiliated Hospital (Brain Disease Center), Henan University of Chinese Medicine, Zhengzhou, Henan, China; 2 The First Clinical Medical College (College of Integrated Chinese and Western Medicine), Henan University of Chinese Medicine, Zhengzhou, Henan, China; 3 Hebi Coal Industry Group General Hospital, Hebi, Henan, China

**Keywords:** digital biomarkers, disease progression, neurofilament light chain, neuroimaging biomarkers, spinocerebellar ataxia

## Abstract

Progressive cerebellar ataxias, including the polyglutamine spinocerebellar ataxias (SCA1, SCA2, SCA3, and SCA6), Friedreich ataxia, and the autosomal recessive and sporadic forms, are rare, clinically heterogeneous neurodegenerative disorders. Disease-modifying therapies, notably antisense oligonucleotides and the approved compound omaveloxolone, are now entering or reaching the clinic, which makes reliable prediction of disease progression a central obstacle to trial success. Rarity, slow and variable decline, and a quantifiable premanifest window together demand sensitive, validated tools to forecast who will progress, how fast, and when symptoms begin. This review synthesizes evidence across five complementary biomarker domains, clinical, imaging, fluid, genetic, and digital, and judges their readiness for forecasting onset and progression. Clinical scales such as SARA capture genotype-specific trajectories but lose sensitivity at the extremes of the disease course. Volumetric, microstructural, and spectroscopic MRI and blood neurofilament light chain change before ataxia onset and predict subsequent decline, whereas repeat length and genetic modifiers set prior risk. Wearable-sensor gait and balance metrics detect change earlier than clinical scales and sharply reduce required sample sizes. We argue that no single modality satisfies every context of use, and that stage-specific, multimodal composites, integrated through harmonized international cohorts and machine learning, offer the most credible path to prognostic enrichment and to shorter, adequately powered, and preventive trials.

## 1 Introduction

The progressive cerebellar ataxias are a group of rare neurodegenerative disorders unified by degeneration of the cerebellum and its connections and divided by cause into autosomal dominant, autosomal recessive, and sporadic forms (1). Clinically they share progressive gait and limb incoordination, dysarthria, and oculomotor disturbance, but they differ widely in associated features, age at onset, and tempo, even among carriers of the same mutation. Among the dominant forms, the polyglutamine spinocerebellar ataxias (SCAs), caused by translated CAG repeat expansions in *ATXN1* (SCA1), *ATXN2* (SCA2), *ATXN3* (SCA3), and *CACNA1A* (SCA6), are the most studied and account for much of the global SCA burden (1, 2). Friedreich ataxia, the most common recessive ataxia, arises from a GAA repeat expansion in *FXN* that lowers frataxin and impairs mitochondrial iron handling (3). Pooled prevalence estimates place dominant ataxias near three and recessive ataxias near three to four per 100,000, although these figures vary with population and ascertainment (4). The genetic landscape continues to widen: biallelic *RFC1* expansion is now recognized as a frequent cause of late-onset sporadic ataxia (5), and a dominantly inherited intronic GAA expansion in *FGF14* explains a substantial fraction of previously unsolved adult-onset cases (6).

For decades, management was limited to symptomatic care, but the field has reached a therapeutic inflection point (7). Gene-silencing antisense oligonucleotides (ASOs) directed at the causative transcript delay or reverse disease features in mouse models of SCA2 and SCA3 (8–10), and several such programs have moved toward or into early-phase human testing (2). In Friedreich ataxia, omaveloxolone became the first agent approved specifically for the disease after a randomized trial showed benefit on a disease-specific examination scale (11), with longer-term comparison against natural-history data supporting a sustained effect (12), and gene and protein-restoration strategies are advancing (13). These developments shift the binding constraint from whether to treat to how to demonstrate treatment effects efficiently.

Three features make that demonstration difficult. The disorders are individually rare, which limits the size of any trial decline on standard clinical measures is slow and variable from person to person and there is a long premanifest interval during which mutation carriers are clinically silent yet may already be accumulating injury (14, 15). Together these features place a premium on biomarkers that can forecast who will progress, how rapidly, and when ataxia will begin. We organize the available tools using the categories of the FDA-NIH Biomarkers, EndpointS, and other Tools (BEST) resource, distinguishing susceptibility or risk, diagnostic, monitoring, prognostic, predictive, and response biomarkers, because a marker useful for one purpose is often unsuited to another (16). This review evaluates five complementary domains, clinical, imaging, fluid, genetic, and digital, against these contexts of use. Our central argument is that prediction adequate for trials will not come from any single marker but from stage-specific combinations integrated across harmonized cohorts, and we give most attention to longitudinal and premanifest evidence, since that is what bears on forecasting.

Two recent syntheses cover adjacent ground and deserve explicit comparison. Klockgether and colleagues review biomarkers in the autosomal dominant spinocerebellar ataxias, organized by biomarker function in the BEST sense, that is, by whether a marker serves a diagnostic, monitoring, prognostic, or response purpose (16). Bernardi and colleagues report a PRISMA-ScR scoping review spanning a wider range of genotypes, including SCA7, SCA17, dentatorubral-pallidoluysian atrophy, SPG7, and fragile X-associated tremor/ataxia syndrome, and treating electrophysiology as a separate domain; its stated objective was to map the breadth of available evidence and identify gaps rather than to assess comparative effectiveness (17). The present review takes a third axis. We organize the evidence by disease stage crossed with context of use, asking not what each marker measures but at which point in the disease course it earns a place in a trial, and we judge the domains comparatively rather than cataloguing them. We then carry that comparison into the currency that constrains trial design, namely the number of participants and endpoint requires, and we state the design assumptions behind every such figure so that readers can see where the comparisons hold and where they do not. Finally, we mark the genotype boundary of the resulting framework rather than implying general applicability, since the longitudinal evidence supporting it comes almost entirely from the polyglutamine SCAs and Friedreich ataxia. A reader wanting a comprehensive map of what has been measured in which ataxia is better served by Bernardi and colleagues, and a reader wanting a function-based account within the dominant SCAs by Klockgether and colleagues. Our aim is narrower and more evaluative: to say which markers can be relied on to forecast onset and progression, at which stage, and at what cost in trial size.

A feature that distinguishes these diseases from many neurodegenerative disorders is that the population at risk can often be identified before symptoms appear. Predictive genetic testing detects mutation carriers years or decades before ataxia begins, which creates both an opportunity, the chance to intervene before irreversible loss, and a requirement, the need for markers that are informative while carriers are still asymptomatic (1). The same interval has been used to assemble prospective cohorts of at-risk individuals whose conversion to manifest disease can be observed directly, turning the premanifest phase from a theoretical construct into a measurable study population that runs through the sections below (18).

## 2 Clinical biomarkers and outcome assessments

Clinician-rated severity scales remain the regulatory backbone of ataxia trials, and the Scale for the Assessment and Rating of Ataxia (SARA) is the de facto standard (15). SARA scores gait, stance, sitting, speech, and limb-kinetic tasks, and it correlates with disability across genotypes. Complementary instruments capture what it omits: the Inventory of Non-Ataxia Signs (INAS) records the pyramidal, extrapyramidal, and peripheral features that accompany ataxia, while performance-based timed measures and composite functional indices add granularity at the milder end of the range (15). In Friedreich ataxia the modified Friedreich Ataxia Rating Scale (mFARS) serves the equivalent role, and its formal test–retest reliability, including the upright-stability subscore that tracks ambulation, is now established (19).

Large natural-history cohorts have defined how these scales move over time. Two-year and long-term follow-up of the European cohort showed that SARA progression is genotype-specific, fastest in SCA1, intermediate in SCA2 and SCA3, and slowest and partly non-linear in SCA6, with the determinants of faster decline differing by genotype (Table 1) (20, 21). Friedreich ataxia progresses at a rate that depends strongly on age at onset and ambulatory status, so that the axial, posture-and-gait items of the mFARS dominate decline in still-ambulatory patients (Table 1) (22). These patterns matter for trial design because a fixed scale behaves like a different instrument depending on the genotype and stage to which it is applied.

**Table 1:** Genotype-specific natural-history progression of the common cerebellar ataxias.

Disorder (gene)	Expansion /Mutation	Annual progression (scale)	Predictors of faster progression	References
SCA1 (*ATXN1*)	CAG	SARA +2.11/year	Longer CAG repeat; older age at inclusion	(21)
SCA2 (*ATXN2*)	CAG	SARA +1.49/year	Earlier age at onset; lower baseline SARA	(21)
SCA3 (*ATXN3*)	CAG	SARA +1.56/year	Longer disease duration at inclusion	(21)
SCA6 (*CACNA1A*)	CAG	SARA +0.80/year (non-linear, slower early)	Lower baseline SARA	(20, 21)
Friedreich ataxia (*FXN*)	GAA	mFARS change varies with onset age and ambulation	Earlier onset; longer shorter-allele GAA; ambulatory axial (upright-stability) items	(22)

SARA, scale for the assessment and rating of Ataxia; mFARS, modified Friedreich Ataxia rating scale. Gene symbols are italicized.

SARA is also not uniformly sensitive across that range. An item-level analysis of more than 800 carriers found that gait, stance, and certain limb items carry most measurable change early, whereas other items contribute mainly once disease is advanced, producing floor and ceiling effects that blunt responsiveness exactly where trials most need it, in early and premanifest disease (23). This is a central limitation of clinician-rated endpoints: they are anchored to overt motor signs and therefore lose traction in the premanifest window that disease-modifying and preventive trials aim to address.

Patient-reported outcomes add a perspective examiner scales cannot. Longitudinal data show that activities-of-daily-living and health-status measures decline measurably but with lower responsiveness than SARA, so they are better suited to demonstrating relevance than to powering short trials (24). Health-related quality of life in the SCAs is driven not only by ataxia severity but also by depression and other mental-health symptoms, which are common and partly independent of motor stage (25, 26). Because depressive symptoms also predict conversion and progression, capturing them serves both ethical and statistical purposes.

Two further clinical domains carry predictive signal. Cognitive-affective change is measurable with the cerebellar cognitive affective, or Schmahmann, syndrome scale (27), and executive and attentional deficits emerge in several genotypes and contribute to disability that motor scales do not register, a non-motor burden patients frequently rank as central. Quantitative oculomotor and electrophysiological testing, particularly saccadic slowing and reduced sensory nerve amplitudes in SCA2, can mark disease before ataxia is manifest (28), so including brief cognitive and oculomotor assessment in a trial battery widens the range of treatment effects that can be detected. Realizing the value of any of these measures across small, geographically dispersed cohorts depends on harmonization. The Ataxia Global Initiative has issued consensus recommendations defining minimal and extended outcome-assessment datasets and calling for evidence on sensitivity to change and on patient-meaningful thresholds (29). Registry and cohort efforts that apply such standards, including multi-site natural-history studies that now incorporate the recently defined *RFC1* and *FGF14* ataxias and dedicated platforms for recessive and sporadic forms, are extending these measures beyond the classic genotypes and providing the denominator needed to judge whether a candidate endpoint behaves consistently across sites and populations (30–32).

Each scale embodies a trade-off. The International Cooperative Ataxia Rating Scale is more granular but longer and more prone to inter-rater variability; the Brief Ataxia Rating Scale is faster but coarser; and performance-based composites built from timed walking, finger-to-nose movement, and speech tasks capture the speed rather than the amplitude of incoordination (15). For trials, the practical question is not which scale is best in the abstract but which is most responsive and least variable in the specific genotype and stage under study, and that question can only be answered with longitudinal data.

## 3 Imaging biomarkers

Structural magnetic resonance imaging (MRI) detects and tracks the degeneration that clinical scales infer indirectly, and it does so earlier and with greater statistical efficiency. Volumetric measures of the pons and cerebellum change faster than SARA over one year in SCA1, and the most responsive imaging metrics achieve larger standardized effects than the clinical scale (Table 2) (33). Brainstem and striatal volume loss is detectable within a single year and predicts subsequent motor decline (34). Beyond simply tracking change, baseline imaging carries prognostic weight, since the degree of regional atrophy at entry forecasts the pace of later decline. The practical implication, set out in Table 2, is that an imaging endpoint should reach significance in a smaller sample than SARA. We note that the SCA1 study reports effect sizes rather than a formal sample-size calculation, so the magnitude of that advantage is inferred from the effect-size ratio and not taken from the source.

**Table 2:** Responsiveness of candidate progression biomarkers relative to the standard clinical scale, with source design assumptions and a recomputation under common assumptions.

Biomarker (modality)	Cohort	Change and effect size	Sample size in source	Recomputed n per arm	References
Pontine volume (MRI volumetry)	SCA1, early to moderate (SARA 0–14); 16 patients, 21 controls; 3 years, 1.5-year intervals	−2.6 ± 1.2%/year; effect size −2.13 ᵃ (SARA +0.60 ᵃ)	Not reported ᵈ	≈ 14 (SARA ≈ 175)	(33)
Pontine tNAA/mIns (MRS)	SCA1, as above; includes 1 premanifest carrier	−3.9 ± 4.6%/year; effect size −0.84 ᵃ	Not reported ᵈ	≈ 88 (SARA ≈ 175)	(33)
SIBA (digital, wearable)	SCA1, 2, 3, 6, ambulatory; 258 patients, 100 controls; validation 53 patients; progression analysis 35–38; 1 year	0.59 versus SARA 0.11 ᵇ	171 versus 1,491 participants in total; 50% slowing, 80% power, 5% significance level	Not computable ᵇ	(35)
Lateral step deviation (digital gait)	SCA2, early; 23 carriers, 9 pre-ataxic; 1 year	*r* = 0.78, *p* = 0.0001 ᶜ; SARA unchanged (*p* = 0.67)	*n* = 43 per arm (37 in the ataxic subgroup); 50% slowing, 80% power, two-sided alpha 0.05	Not computable ᶜ	(62)
Plasma NfL (fluid)	Polyglutamine SCAs, preataxic to manifest	Predicts atrophy and progression; little within-person change over 1–2 years	Not reported ᵉ	Not applicable	(45, 51)

MRS, magnetic resonance spectroscopy; NAA, N-acetylaspartate; NfL, neurofilament light chain; SARA, Scale for the assessment and rating of ataxia; SIBA, score of integrated balance in Ataxia.

Effect-size metrics are not interchangeable: ᵃ standardized response mean, mean percent change divided by the standard deviation of percent change; ᵇ the ratio of the group-average regression slope to the average standard error of the individual slope estimates, which is scaled by a standard error rather than a between-subject standard deviation; ᶜ matched-pairs rank biserial correlation from a Wilcoxon signed-rank test, bounded 0 to 1. ᵈ Source reports effect sizes only, with no power calculation; the expectation of a smaller required sample is our inference. ᵉ Proposed for prognostic enrichment rather than monitoring. Source sample sizes are reproduced as published and are not comparable across rows. Recomputation was possible only for endpoints reporting a standardized response mean, and applies one common set of assumptions: two-arm design with 1:1 allocation, one-year change endpoint, 50% reduction targeted, 80% power, two-sided alpha 0.05, no dropout. Recomputed values are a benchmark, not a correction, and inherit the imprecision of the small cohorts from which the variances derive.

Microstructural and neurochemical methods push sensitivity into the premanifest range. Diffusion MRI of the cerebellar peduncles, brainstem, and their constituent fiber tracts separates carriers from controls with high accuracy and changes during the preataxic phase of SCA3, and fixel-based analysis of these tracts performs strongly as a marker of disease state (35, 36). These changes are not diffuse but follow the anatomy of each disease, affecting the peduncles, the brainstem, and sensory pathways such as the medial lemniscus, and modeling them against the estimated time to onset shows that microstructural change is already underway during the premanifest interval (37). In a dedicated preataxic cohort, neurochemical and microstructural endpoints discriminated SCA1 and SCA3 carriers from controls with excellent accuracy, in several cases outperforming volumetry at the earliest stages (36). Magnetic resonance spectroscopy (MRS) adds biochemical specificity: the ratio of total N-acetylaspartate, an index of neuronal integrity, to myo-inositol, a glial marker, shifts in the pons before clinical onset and correlates with severity, so structural and metabolic signals reinforce one another (33, 38).

Genotype-specific imaging signatures are emerging. Pontine and cerebellar atrophy is present in preclinical SCA2 and tracks with severity (39), and structural changes there relate to clinical and neurophysiological phenotype, including sleep microstructure (40). Coordinated multimodal follow-up of preataxic and early SCA2 and SCA7 carriers has quantified one-year brainstem and cerebellar volume change alongside clinical and fluid markers, demonstrating that imaging can anchor short premanifest studies (41).

Several measures capture disease in tissues that standard scales ignore. In Friedreich ataxia, quantitative susceptibility mapping (QSM) shows progressive iron accumulation in the dentate nucleus whose rate tracks disease stage, with atrophy and iron change following distinct time courses (42). Cervical spinal cord cross-sectional area is reduced from early in the disease and declines progressively, reflecting loss in the dorsal columns and corticospinal tracts whose degeneration underlies the sensory and motor signs; this was established in a large multi-site analysis that also illustrates how harmonized consortia overcome single-center sample limits (43). Retinal optical coherence tomography (OCT) provides an accessible window on neurodegeneration, since the layered retina allows specific neuronal populations to be quantified non-invasively: outer-retinal and ganglion-cell thinning correlate with severity and duration in SCA3 (44), and progressive photoreceptor loss is a defining feature of SCA7 (40).

Imaging is the most mature source of prognostic and monitoring biomarkers in these diseases. The orderly spread of atrophy allows patients to be assigned to prognostically meaningful stages that can enrich trials (45), and a recurring theme across these methods is that the lowest measurement noise, not merely the earliest signal, is what delivers statistical efficiency, since automated, reproducible quantification reaches significance in smaller samples than an examiner rating can (34). Multi-center use has nonetheless been limited by differences in scanners, sequences, and analysis pipelines. The Ataxia Global Initiative MRI working group has therefore issued recommendations on acquisition and harmonized endpoints intended to make volumetric, diffusion, and spectroscopic measures usable as trial outcomes across sites, and harmonization is treated as a precondition rather than a refinement (46). In our assessment, structural volumetry is ready now for prognostic enrichment and monitoring, whereas diffusion and spectroscopic measures, although more sensitive preataxically, still require multisite validation before they can serve as primary endpoints.

## 4 Fluid biomarkers

Blood-based markers of neuronal injury have reshaped biomarker prospects in many neurodegenerative diseases, and neurofilament light chain (NfL) is the leading example. NfL is released on axonal damage and can be measured in cerebrospinal fluid (CSF) and, with fourth-generation assays, reliably in blood; successive assay generations, from immunoblot through enzyme-linked methods to electrochemiluminescence and single-molecule arrays, progressively lowered detection limits until venous sampling replaced lumbar puncture as the practical route. Its concentration rises with age in healthy people, so interpretation requires age-adjusted references (47). These properties make NfL attractive as a minimally invasive marker but also impose analytical and interpretive caveats that recur throughout the ataxia literature.

Across the genetic ataxias, blood NfL is elevated and tracks proximity to onset. A meta-analysis spanning multiple genotypes found higher blood NfL in carriers than controls, with values increasing as clinically manifest disease approaches and a clear age dependence (48). In SCA3, NfL is raised years before the predicted onset, the same elevation is reproduced in knock-in mice and precedes Purkinje-cell loss, and serum and CSF concentrations are tightly correlated (49, 50). This convergence of human and animal evidence is unusually strong and supports NfL as a susceptibility and prognostic marker in the premanifest window.

A nuance tempers that enthusiasm. In several polyglutamine SCAs, plasma NfL is high but changes little over one to 2 years despite measurable clinical and imaging progression, so within-person longitudinal NfL is a weak monitoring marker over short intervals. Its value is prognostic rather than dynamic: a high baseline concentration predicts subsequent atrophy and clinical worsening and can therefore enrich trials for fast progressors (51). NfL has separately been proposed as a response biomarker across SCA subtypes, on the reasoning that it should index ongoing neurodegeneration and so fall if a therapy slows it, but the data needed to support that use are not yet available, and the weak short-term within-person change described above is precisely what makes the case unproven (16). Glial fibrillary acidic protein (GFAP) and phosphorylated neurofilament heavy chain extend the panel. GFAP and NfL together discriminate SCA7 carriers and correlate with severity, pointing to a reactive astrocytic component alongside axonal loss (52), and NfL marks neurodegeneration in ataxia telangiectasia (53).

Friedreich ataxia illustrates why disease-specific calibration is essential. There, blood NfL is elevated in childhood but declines with age, converging toward control values in the third to fourth decade, the opposite of the age-related rise seen in unaffected people, while tau is elevated across ages (54, 55). NfL concentrations are also stable over a year in this disease. A marker that falls with age in one ataxia and rises in another cannot be interpreted without genotype-specific and age-specific reference data, a point with direct consequences for trials that enroll across wide age ranges.

A distinct and increasingly important category is the response biomarker that reports target engagement. The disease protein itself can be quantified: polyglutamine-expanded ataxin-3 is measurable in peripheral blood mononuclear cells and correlates with clinical parameters in SCA3, offering a candidate pharmacodynamic readout for *ATXN3*-lowering therapies (56). In Friedreich ataxia, assays that quantify mature and extra-mitochondrial frataxin in blood provide a direct measure for therapies that aim to restore the deficient protein (57). Markers that integrate across domains are also appearing: in SCA3, cognitive impairment emerging after ataxia onset is associated with lower regional brain volume and higher NfL, linking fluid, imaging, and clinical change (58). The principal limitations of fluid markers remain biological non-specificity, age dependence, and the absence of cross-platform assay standardization.

## 5 Genetic biomarkers

Genetic information is the earliest available predictor, present from birth and stable thereafter. In the polyglutamine SCAs, the length of the expanded CAG repeat is the principal determinant of age at onset and influences the rate of progression, an effect embedded in the genotype-specific trajectories summarized in Table 1 (21). In Friedreich ataxia, the length of the shorter GAA allele correlates with earlier onset and faster decline, which helps explain the heterogeneity of progression on which trial stratification depends (3, 22).

Repeat length is necessary but not sufficient to predict the individual course, and attention has turned to modifiers. Somatic instability of the repeat and variation in DNA-repair pathways modify onset and progression in Huntington disease, the best-powered polyglutamine model, where repair-gene variants shift motor and cognitive milestones, and the same mechanisms are strong candidates in the SCAs (59). Within the repeat itself, configuration matters: interrupting sequences alter the relationship between length and phenotype, so simple repeat sizing can mislead (2). These observations argue for treating the genotype as more than a single number.

Because repeat length sets risk without timing it precisely, the most useful genetic prediction combines the mutation with age and other dynamic information. In carriers not yet ataxic, models that integrate repeat length with age and baseline clinical and biological features estimate the probability and timing of conversion (18, 60). The genetic differential has also broadened in ways that change practice: biallelic *RFC1* expansion accounts for a large share of late-onset sporadic ataxia, frequently with vestibular and sensory involvement (5), and intronic *FGF14* GAA expansion explains a sizable fraction of otherwise unexplained adult-onset cases and may show repeat-length-dependent features (6). The limitation of all genetic markers is intrinsic: they define static risk and must be paired with a dynamic modality to track an individual over time.

Somatic expansion, the tendency of the repeat to lengthen further in vulnerable neurons across the lifespan, is increasingly seen as a driver of the timing of neurodegeneration rather than a passive correlate, and variants in the DNA mismatch-repair machinery that govern this instability shift the age at which disease milestones are reached (59). Sequence interruptions within the tract, such as the loss of stabilizing units in *ATXN1*, alter both the stability and the toxicity of the expansion, so two carriers with the same nominal repeat count can follow materially different courses, which limits the precision of repeat length used on its own (2).

The phenotypes of the newly recognized repeat disorders carry prognostic texture of their own. Biallelic *RFC1* disease often combines cerebellar, vestibular, and sensory features in a slowly progressive syndrome (5), whereas intronic *FGF14* expansion can present with episodic symptoms before becoming steadily progressive and may respond to symptomatic treatment, which makes early genetic identification clinically actionable (6). Across all of these, the genotype predicts the average carrier well but the individual poorly, so accurate forecasting pairs the static repeat measure with a dynamic marker of current disease state (60).

Carrying this forward, onset estimated from repeat length and age lets carriers be placed on a shared timeline anchored to their predicted onset, which is what allows individuals with different mutations and ages to be combined in a single analysis and compared on a common scale (18).

## 6 Digital biomarkers

Wearable-sensor measures of movement promise frequent, objective, and sensitive quantification of the motor features that define these diseases. Inertial sensors recording gait reveal that the variability of stepping, rather than average speed, best distinguishes ataxia. The reason is mechanistic: cerebellar dysfunction degrades the consistency of movement more than its average velocity, so metrics that quantify step-to-step irregularity capture the core deficit and separate prodromal carriers from controls before mean performance has clearly declined (Table 2) (61).

The decisive advantage of digital measures is sensitivity to change over short intervals. In early and preataxic SCA2, a digital gait metric captured one-year progression that the clinical scale did not, implying a marked reduction in the sample size required for a trial of given duration (Table 2) (62). A composite balance score derived from wearable sensors across SCA1, SCA2, SCA3, and SCA6 correlated with clinical severity, showed greater one-year responsiveness than SARA, and would require far fewer participants to power a progression trial (Table 2) (35). ese are among the largest efficiency gains reported for any candidate endpoint in the ataxias, although the two figures were derived in different genotypes, at different stages and under different statistical assumptions, and should not be read as directly comparable.

Capturing movement in daily life, rather than in the clinic, adds ecological validity. Laboratory walking captures capacity under ideal conditions, whereas sensors worn during daily life capture performance, what a person actually does, and the two can diverge. Body-worn sensors recording real-life walking yield ataxia-specific variability measures that correlate with severity and provide Class I evidence of validity, with the largest effects seen during everyday activity (63). In Friedreich ataxia, accelerometer-based measures of habitual activity track the disease over time (64). Continuous home monitoring also dampens the influence of single-day performance fluctuation that limits in-clinic testing.

The same logic extends beyond gait. Speech is an attractive target because it is cerebellar, easily recorded, and amenable to automated acoustic analysis; in SCA2, reduced agility on rapid syllable-repetition tasks and slowed syllable rate appear before ataxia is clinically manifest and worsen with the disease, marking the prodromal phase through the voice alone (65). Digital-motor outcomes also transfer to neighboring degenerative diseases: in hereditary spastic paraplegia type 7, multicenter wearable measures captured patient-relevant change, showing that the approach generalizes across the broader ataxia and spastic-ataxia spectrum (66).

Digital endpoints are maturing quickly, but their path to regulatory qualification requires demonstrating measurement reliability across devices, firmware versions, and home environments, defining clinically meaningful change in terms patients recognize, and guarding against the access inequities that remote technologies can introduce; multicenter studies in related spastic ataxias indicate these steps are achievable (66). In our view, wearable gait and balance metrics are the most promising near-term complement to clinical scales for early-stage and premanifest trials, precisely because their sensitivity is greatest where clinician-rated scales are weakest.

## 7 Integrating biomarkers across modalities

**Figure 1:**
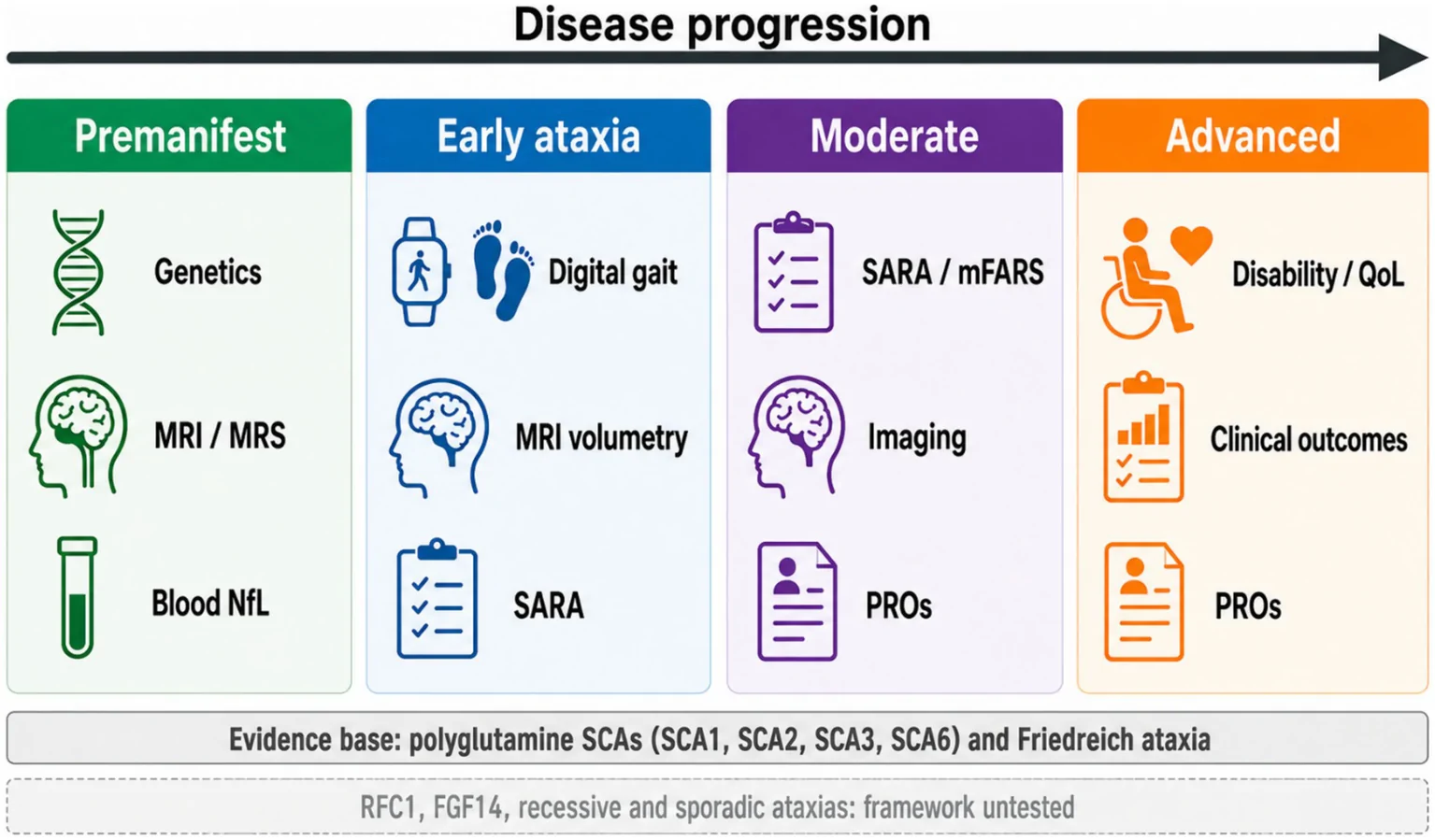
Stage-specific biomarker framework for predicting progression in progressive cerebellar ataxias. Biomarker domains are mapped against the disease course, with shaded blocks marking where each performs best. No domain spans the whole course: Genetics sets risk but never tracks change, imaging and fluid markers become informative before onset, digital measures are most responsive as ataxia emerges, and clinical and patient-reported outcomes track disability once it accrues. Endpoint choice should therefore follow disease stage rather than any general ranking. Supporting evidence comes from the polyglutamine SCAs and Friedreich ataxia; transportability to RFC1, FGF14 and the recessive and sporadic forms is not yet established. MRS, magnetic resonance spectroscopy; NfL, neurofilament light chain; SARA, Scale for the Assessment and Rating of Ataxia.

No single biomarker satisfies every context of use, and the domains reviewed here have complementary strengths and failure modes. Genetic markers define risk but not timing; clinical scales anchor relevance but lose sensitivity at the extremes; imaging and fluid markers move before symptoms but, respectively, demand harmonization and resist short-term within-person change; digital measures are sensitive and frequent but not yet qualified. Mapping each modality to its best-supported context of use and to the disease stage at which it performs best (Table 3) makes the case for deliberate combination rather than competition. Because biomarker performance varies across the disease course, a stage-specific framework for selecting prognostic and monitoring biomarkers is summarized in Figure 1.

**Table 3:** Biomarker modalities mapped to FDA-NIH BEST contexts of use and optimal disease stage.

Modality (Example marker)	BEST context of use	Optimal disease stage	Trial-readiness	References
Clinical (SARA, INAS)	Monitoring	Early to moderate	Established (primary endpoint)	(21, 30)
Imaging volumetry (pons, cerebellum)	Monitoring; prognostic	Preataxic to advanced	Advanced; harmonization ongoing	(33, 38)
Imaging diffusion / MRS	Prognostic; monitoring	Preataxic to early	Emerging (multisite)	(36, 38)
Fluid blood NfL	Prognostic; susceptibility	Preataxic to early	Promising; needs standardization	(48, 49)
Fluid mutant protein (*ATXN3*)	Response (target engagement)	On treatment (all stages)	Assay-dependent; emerging	(9, 56)
Genetic repeat length (and modifiers)	Susceptibility; prognostic	Preataxic (static)	Established (enrichment)	(18, 21)
Digital wearable gait / balance	Monitoring; prognostic	Preataxic to early	Emerging; maturing rapidly	(35, 62)

BEST, Biomarkers, EndpointS, and other Tools; INAS, Inventory of Non-Ataxia Signs; MRS, magnetic resonance spectroscopy; NfL, neurofilament light chain; SARA, scale for the assessment and rating of ataxia. Gene symbols are italicized.

Prediction should therefore be stage-stratified. In the premanifest period, static genetic risk combined with neurochemical or microstructural MRI and blood NfL gives the most credible estimate of whether and when ataxia will begin (38, 51). As ataxia emerges, digital gait and balance metrics and volumetric MRI become the most responsive monitors of early change (35, 62). In moderate to advanced disease, clinician-rated and patient-reported measures retain the clearest link to disability even as some instrumental markers approach ceiling. Matching the marker to the stage is the single most actionable principle to emerge from the evidence.

The framework set out above rests on a narrow evidence base. Almost all of the longitudinal, premanifest and multimodal data reviewed here come from the polyglutamine SCAs and Friedreich ataxia, while the recently characterized late-onset repeat ataxias have been studied along quite different lines. The largest longitudinal series in GAA-FGF14 ataxia describes progression of about 0.29 SARA points per year, roughly a seventh of the rate in SCA1, with serum NfL not elevated relative to controls (67). In biallelic RFC1 disease, a recent cohort reports approximately 1.2 SARA points per year, and repeat conformation is itself heterogeneous, with AAGGG and ACAGG configurations differing in phenotype and possibly in tempo (68, 69). The recessive and sporadic forms are currently served by registry and cohort infrastructure rather than by validated longitudinal endpoints (30, 31).

These are not only gaps in the evidence but reasons the framework may not transport. The premanifest stage on which the earliest predictions depend presupposes an identifiable at-risk population, which predictive testing supplies in the dominant polyglutamine SCAs but which a recessive, late-onset disorder such as RFC1 disease does not. The sample-size arithmetic of Table 2 is calibrated on polyglutamine progression rates, so an endpoint powered against SCA1 decline would be substantially underpowered against a disorder progressing several times more slowly. Most directly, the fluid marker that looks most transportable does not transport: serum NfL is not raised in GAA-FGF14 ataxia, consistent with its slow tempo and limited extracerebellar involvement, so a trial designed around a rising injury marker would have no signal to read. Late-onset disease adds a further confound, since concurrent neurodegenerative pathology can dominate individual trajectories in a way that is uncommon in young-onset polyglutamine disease (67). The framework should therefore be read as established for the polyglutamine SCAs and Friedreich ataxia and as a hypothesis elsewhere. Extending it will require genotype-specific work rather than extrapolation: cohorts sized for slower progression, marker selection that does not assume axonal injury of polyglutamine magnitude, and staging schemes for the recessive and sporadic forms that do not depend on a premanifest window at all. Digital measures may prove the most portable, since gait variability indexes cerebellar dysfunction directly rather than through a disease-specific molecular process, but this is a prediction to be tested.

**Figure 2:**
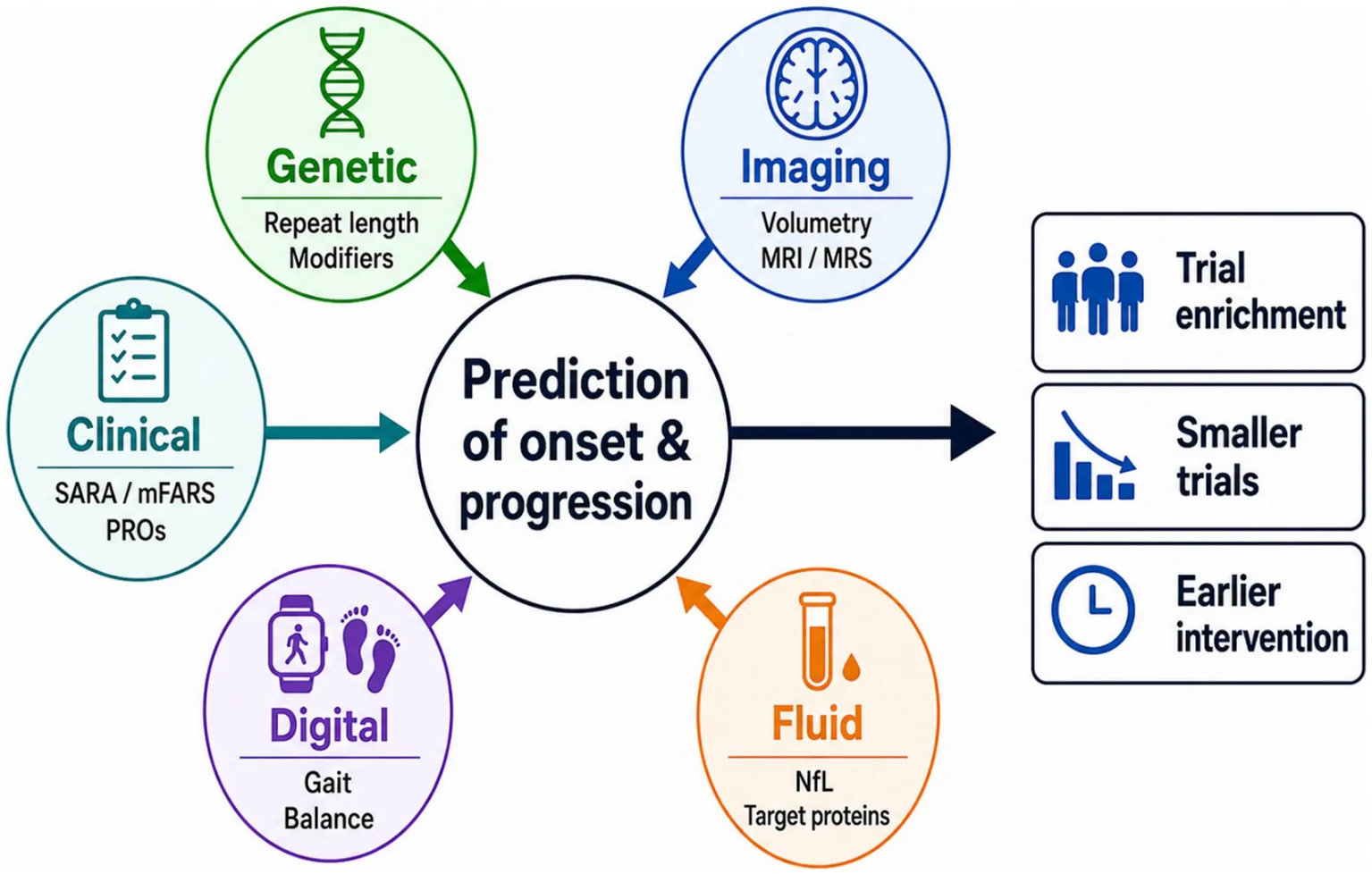
Multimodal biomarker integration for prediction and trial enrichment in progressive cerebellar ataxias. The five biomarker domains feed an integration layer in which harmonized cohort data and machine-learning models combine static risk with dynamic measures of current disease state, yielding individualized estimates of onset and rate. These support prognostic enrichment, which selects participants most likely to progress within the trial window, and endpoint selection, which pairs that enrichment with the most responsive monitoring measure for the stage. NfL, neurofilament light chain.

Formal multimodal models outperform any single marker. In SCA1 and SCA3, models combining clinical, imaging, and fluid variables predict progression and conversion, with different variables governing each, so that the predictors of conversion are not the predictors of post-onset progression (60). Data-driven brain-atrophy staging in SCA3 stratifies patients for prognosis and would enrich trials for those most likely to progress (45). Coordinated cross-modality follow-up confirms that imaging, fluid, and clinical changes can be measured together in the same carriers over short intervals, the prerequisite for building and validating such models (41). The proposed multimodal integration framework is illustrated in Figure 2.

The payoff is statistical and practical. Because the most sensitive imaging and digital measures reach significance in smaller samples than clinical scales (Table 2), pairing a prognostic enrichment marker with a sensitive monitoring endpoint can make an otherwise infeasible trial achievable in a rare disease. The size of that gain should be read with care. The estimates in Table 2 come from studies that differ in genotype, disease stage, follow-up interval, effect-size definition, and the treatment effect, power and alpha assumed in each calculation, so they document what each endpoint claimed for itself rather than establishing a common ranking. A properly comparable estimate would require recomputing sample sizes for every candidate endpoint from a single harmonized cohort under one set of assumptions, which is not yet possible with published data and is a concrete task for the multinational consortia discussed below. Realizing this depends on infrastructure: harmonized multinational cohorts and registries with shared protocols, including the recently characterized non-polyglutamine repeat ataxias, supply the standardized longitudinal data that model-building and qualification require (29–31, 46). The biomarker framework that distinguishes contexts of use provides the common language for assembling these pieces into trial-ready packages (16).

To make a limited comparison possible we recomputed sample sizes under a single set of assumptions, given in the footnote to Table 2. Recomputation was possible only where a source reported a standardized response mean, which is to say within the one SCA1 cohort in which volumetry, spectroscopy and SARA were measured together. On that common footing, pontine volume would require approximately 14 participants per arm, pontine total N-acetylaspartate to myo-inositol approximately 88, and SARA approximately 175. This is the one strictly like-for-like comparison the published data support, because the three endpoints share a cohort, a follow-up interval and a computational method. The digital endpoints cannot be placed on the same footing, and the reason is instructive rather than incidental. The digital balance composite reports an effect size defined as the group-average progression slope divided by the average standard error of the individual slope estimates, and the SCA2 gait measure reports a nonparametric rank biserial correlation bounded between zero and one. Neither is a standardized response mean, so neither can be read against a value such as −2.13, and neither yields to the same power calculation. The published sample sizes for these endpoints also rest on cohort-specific features that do not generalize; in the digital balance study, for instance, the comparator figure of 1,491 reflects a SARA progression rate of 0.22 points per year, considerably slower than reported elsewhere and attributed by the authors to the recruitment of early, ambulatory participants. The practical implication is that responsiveness claims across modalities cannot currently be ranked, and that any future comparison will require a common effect-size definition applied within a harmonized cohort.

The complementarity is structural rather than incidental: a marker stable enough to define lifelong risk is by construction insensitive to change over months, while a marker sensitive enough to track monthly change is by construction noisy as a one-time risk estimate, so no single measurement can serve both purposes and a panel necessarily outperforms any element of it (16). The finding that conversion and post-onset progression are governed by different variables sharpens the point, since a marker chosen to enrich a prevention trial for imminent converters need not be the marker that best tracks decline once disease begins (60).

Translating these principles into practice means matching the endpoint to the trial question at each stage, so that a prevention study reads out on a microstructural or neurochemical marker while enriching on genetic risk, an early-symptomatic study reads out on a digital or volumetric marker while enriching on a baseline injury marker, and a study in established disease keeps a clinician or patient-reported primary endpoint anchored to disability (38, 51, 62).

**Figure 3:**
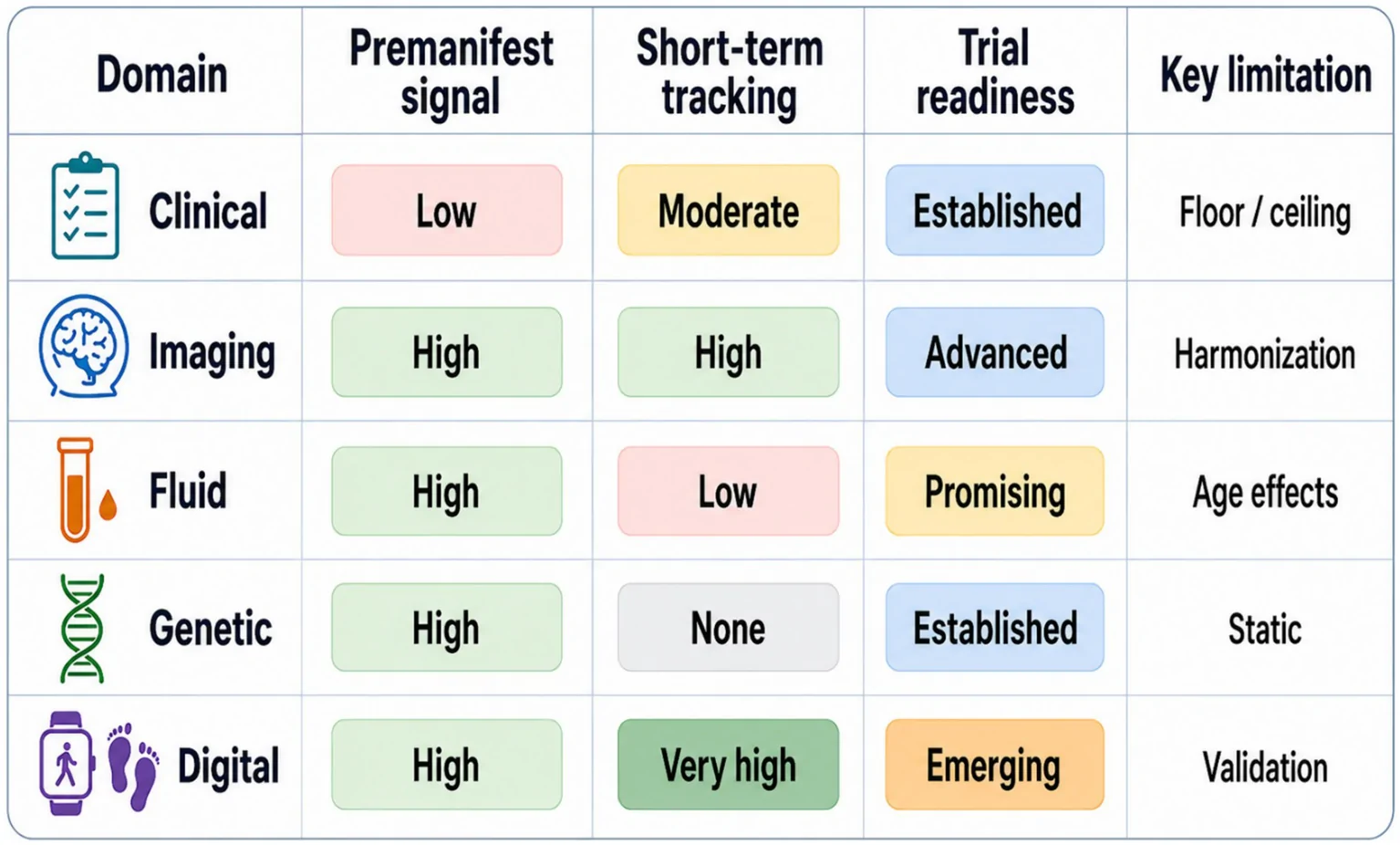
Comparative utility of biomarker domains in progressive cerebellar ataxias. The five domains are compared across the attributes that determine fitness for trial use: Sensitivity to premanifest and early change, short-term responsiveness, prognostic value, trial readiness, and burden. Genetic markers rate highest for early availability and lowest for responsiveness; clinical scales highest for regulatory acceptance and lowest for early sensitivity; imaging and digital measures fall between, offering the greatest short-term responsiveness while harmonization and qualification remain incomplete. Ratings are a qualitative summary of the evidence in sections 2 to 6, not the output of a formal scoring instrument.

None of this is achievable at single centers, where the rarity of each genotype caps achievable sample sizes; it depends on multinational consortia that pool standardized longitudinal data, apply common protocols across imaging, fluid, digital, and clinical domains, and increasingly include the recently characterized repeat ataxias (30, 31, 46). Machine-learning models trained on such pooled data can compress many features into a single individualized estimate of onset or rate, but their value depends on the quality and representativeness of the training data and on prospective rather than retrospective validation (29). The relative utility, trial readiness, and major limitations of each biomarker domain are summarized in Figure 3.

## 8 Challenges, future directions, and conclusion

Several obstacles stand between the current evidence and routine predictive use. The most pressing is standardization. Fluid assays need cross-platform calibration and age-adjusted, genotype-specific reference ranges before any single threshold can be trusted across laboratories; imaging requires harmonized acquisition and analysis so that volumetric, diffusion, and spectroscopic measures mean the same thing across scanners; and digital tools need demonstrated reliability across devices, firmware versions, and home settings, since a change of sensor or of a manufacturer’s proprietary processing algorithm can shift a derived gait metric without any change in the patient. Passive home monitoring also carries obligations that a clinic visit does not, because continuously collected movement data are identifiable and fall under data-protection rules that differ across the jurisdictions a multinational cohort spans, so consent and data-sharing arrangements need to be settled at protocol design rather than retrofitted. Each modality also needs both analytical validation, showing the measurement is accurate and reproducible, and clinical validation, showing it predicts outcomes that matter. Equity is a parallel concern, and it bears on prediction specifically rather than as a general point of fairness. Most cohorts over-represent populations of European ancestry and well-resourced centers, yet repeat-length thresholds, age-at-onset models, and age-adjusted NfL reference ranges are all calibrated on the population sampled, so a cut-off derived in one group may misclassify in another; the ultra-rare genotypes are affected differently again, since almost no genotype-specific longitudinal data exist for them. Remote digital assessment expands geographic reach but introduces a gradient of its own, because participation depends on owning a compatible device and on stable connectivity, so those excluded from conventional trials by distance from a specialist center may also be least able to join a decentralized one. Predictive tools validated in narrow samples may not transfer, and the ancestry and site composition of the cohorts underlying any published model should be reported as a matter of course.

The same caution applies by genotype as well as by ancestry. As set out in section 7, the stage-specific framework advanced here is supported by evidence from the polyglutamine SCAs and Friedreich ataxia and remains untested in the RFC1, FGF14, recessive and sporadic forms, and we present it as established only for the former.

A further obstacle is regulatory. Novel imaging, fluid, and digital endpoints must pass formal qualification before they can support labeling claims, a process that demands exactly the standardized, prospectively collected, multimodal datasets the field is only now assembling. Preventive trials will additionally require agreement on what counts as a meaningful delay of onset and on the ethical framework for treating asymptomatic carriers, questions that are as much about consensus among regulators, clinicians, and patients as about measurement.

The trajectory of the field points in clear directions. The premanifest window is the natural target, and the convergence of genetic risk, early imaging change, and rising injury markers makes preventive and delay-of-onset trials realistic for the first time. Future prediction will rest on integrated panels that combine a static risk estimate, a sensitive monitoring endpoint, and a response marker confirming target engagement, rather than on any one measurement. Computational models, including machine-learning approaches trained on harmonized multimodal data, are likely to provide individualized estimates of onset and rate, provided they are validated prospectively and across diverse populations. As gene and protein-directed therapies enter the clinic, response biomarkers that report engagement of the causal mechanism will become as important as the progression markers that dominate current practice.

Prediction adequate for clinical trials in the progressive cerebellar ataxias will not come from a single biomarker. It will come from stage-specific, analytically and clinically validated combinations, genetic risk with early imaging and fluid markers in the premanifest period, sensitive digital and volumetric measures as ataxia emerges, and clinician and patient-reported outcomes as disability accrues, assembled within a shared framework of biomarker contexts of use and supported by harmonized international cohorts. The component pieces now exist. The task ahead is to integrate, standardize, and validate them so that shorter, smaller, and earlier trials can reliably detect the disease-modifying effects that are finally within reach.
